# Identification of Critical Regions and Candidate Genes for Cardiovascular Malformations and Cardiomyopathy Associated with Deletions of Chromosome 1p36

**DOI:** 10.1371/journal.pone.0085600

**Published:** 2014-01-15

**Authors:** Hitisha P. Zaveri, Tyler F. Beck, Andrés Hernández-García, Katharine E. Shelly, Tara Montgomery, Arie van Haeringen, Britt-Marie Anderlid, Chirag Patel, Himanshu Goel, Gunnar Houge, Bernice E. Morrow, Sau Wai Cheung, Seema R. Lalani, Daryl A. Scott

**Affiliations:** 1 Department of Molecular and Human Genetics, Baylor College of Medicine, Houston, Texas, United States of America; 2 Department of Molecular Physiology and Biophysics, Baylor College of Medicine, Houston, Texas, United States of America; 3 Institute of Genetic Medicine, Newcastle University, Newcastle upon Tyne, United Kingdom; 4 Department of Clinical Genetics, Leiden University Medical Center, Leiden, The Netherlands; 5 Clinical Genetic Department, Karolinska University Hospital and Institution of Molecular Medicine and Surgery, Karolinska Institutet, Stockholm, Sweden; 6 Department of Clinical Genetics, Birmingham Women’s Hospital, Birmingham, United Kingdom; 7 Faculty of Health and Medicine, University of Newcastle, Callaghan, New South Wales, Australia; 8 Center for Medical Genetics and Molecular Medicine, Haukeland University Hospital, Bergen, Norway; 9 Department of Genetics, Albert Einstein College of Medicine, Bronx, New York, United States of America; Duke University, United States of America

## Abstract

Cardiovascular malformations and cardiomyopathy are among the most common phenotypes caused by deletions of chromosome 1p36 which affect approximately 1 in 5000 newborns. Although these cardiac-related abnormalities are a significant source of morbidity and mortality associated with 1p36 deletions, most of the individual genes that contribute to these conditions have yet to be identified. In this paper, we use a combination of clinical and molecular cytogenetic data to define five critical regions for cardiovascular malformations and two critical regions for cardiomyopathy on chromosome 1p36. Positional candidate genes which may contribute to the development of cardiovascular malformations associated with 1p36 deletions include *DVL1*, *SKI*, *RERE*, *PDPN*, *SPEN*, *CLCNKA*, *ECE1*, *HSPG2*, *LUZP1*, and *WASF2*. Similarly, haploinsufficiency of *PRDM16*–a gene which was recently shown to be sufficient to cause the left ventricular noncompaction–*SKI*, *PRKCZ*, *RERE*, *UBE4B* and *MASP2* may contribute to the development of cardiomyopathy. When treating individuals with 1p36 deletions, or providing prognostic information to their families, physicians should take into account that 1p36 deletions which overlie these cardiac critical regions may portend to cardiovascular complications. Since several of these cardiac critical regions contain more than one positional candidate gene–and large terminal and interstitial 1p36 deletions often overlap more than one cardiac critical region–it is likely that haploinsufficiency of two or more genes contributes to the cardiac phenotypes associated with many 1p36 deletions.

## Introduction

Approximately 1 in 5000 newborns has a terminal deletion affecting chromosome 1p36, making it the most common telomeric deletion in humans [1]. Individuals with terminal 1p36 deletions share a common set of phenotypes that constitute the 1p36 deletion syndrome [2], [3]. These phenotypes include typical craniofacial features, cognitive impairment, behavioral problems, seizures, postnatal growth deficiency, eye/vision problems, hearing loss, cleft palate, cardiovascular malformations, cardiomyopathy and renal anomalies.

The distal critical regions for most 1p36 deletion syndrome phenotypes have been determined to reside within approximately 4 Mb from the 1p telomere [4]. However, non-overlapping interstitial deletions involving the proximal region of 1p36, starting approximately 8 Mb from the 1p telomere, have also been shown to cause many of the phenotypes associated with distal 1p36 deletions including cognitive impairment, seizures, postnatal growth deficiency, cardiovascular malformations and cardiomyopathy [5]. Some individuals have deletions of both the distal and proximal regions of 1p36 [6]. In such cases, the additive effects of haploinsufficiency of genes within both of these regions may account for the observation that individuals carrying larger 1p36 deletions are more severely affected and exhibit more of the features typically associated with 1p36 deletions [7], [8].

Cardiovascular malformations and cardiomyopathy are among the most acutely life-threatening conditions associated with both distal and proximal deletions of 1p36. Heterozygous loss-of-function mutations in *PRDM16*–a gene in the distal portion of 1p36 (2,985,742–3,355,185; hg19)–were recently shown to be sufficient to cause left ventricular noncompaction [9]. Similarly, a heterozygous loss-of-function mutation in *ECE1* (21,543,740–21,672,034; hg19) has been identified in an individual with patent ductus arteriosus, a small subaortic ventricular septal defect, and a small atrial septal defect, suggesting that haploinsufficiency of this proximal 1p36 gene may be sufficient to cause cardiovascular malformations in humans [10]. However, the other genomic regions and dosage-sensitive genes that contribute to the cardiac phenotypes caused by 1p36 deletions have not been clearly defined. This paucity of information makes it difficult for physicians to create individualized medical plans and provide accurate prognostic information to patients and families affected by 1p36 deletions. This is true even though detailed information regarding the extent and location of an individual’s 1p36 deletion can be readily obtained on a clinical basis. Defining the cardiac-related regions and genes on 1p36 will not only allow physicians to provide improved medical care, but is also a prerequisite to understanding the molecular mechanisms that underlie the development of 1p36-related cardiovascular malformations and cardiomyopathy. Elucidating these molecular mechanisms may lead to the development of novel preventative and/or therapeutic interventions for cardiovascular disorders.

Using clinical and molecular cytogenetic data from individuals with isolated 1p36 deletions defined by array-based copy number analysis, we have identified five non-overlapping critical regions for cardiovascular malformations and two non-overlapping critical regions for cardiomyopathy on chromosome 1p36. A bioinformatic analysis of each of these cardiac critical regions revealed at least one positional candidate gene whose deletion may contribute to the development of cardiac phenotypes based on human studies and/or animal models. In some cases, haploinsufficiency of two or more such genes may contribute to the cardiac phenotypes associated with a 1p36 deletion. This is particularly likely in the case of large terminal and interstitial deletions that overlap more than one cardiac critical region.

## Materials and Methods

### Ethics Statement

These studies were performed under research protocols approved by the institutional review board of Baylor College of Medicine. All clinical investigations were conducted according to the principles expressed in the Declaration of Helsinki. Written informed consent was obtained from parents or guardians on behalf of study participants all of whom were minors/children at the time of enrollment.

### Patient Identification and Accrual

Molecular and clinical data from individuals with isolated 1p36 deletions associated with cardiovascular malformation and/or cardiomyopathy were collected from four sources. The first source consisted of data from 33,566 de-identified individuals referred to the Medical Genetics Laboratories at Baylor College of Medicine for array-based copy number analysis. Only data from individuals with isolated 1p36 deletions and an indication for testing that included a cardiovascular malformation or cardiomyopathy were included in this study.

The second source consisted of data from patients with isolated 1p36 deletions confirmed by array-based copy number analyses who were accrued from a group of individuals receiving care at Texas Children’s Hospital in Houston, TX, USA and by self-referral. In these cases, informed consent was obtained under a protocol approved by the institutional review board of Baylor College of Medicine after which clinical and molecular cytogenetic data was obtained from a review of the medical record and correspondence with the individual’s parents/family members.

The third source consisted of data from individuals with isolated 1p36 deletions associated with cardiac phenotypes who were listed in the Database of Chromosomal Imbalance and Phenotype in Humans using Ensembl Resources (DECIPHER; http://decipher.sanger.ac.uk/). In each case, representatives of the contributing institution–where consent for the submission of clinical and molecular cytogenetic data were obtained–were contacted and given an opportunity to provide further details of the phenotypes present in their patients.

The final source was a literature review conducted to identify additional individuals whose cardiac phenotypes were caused by isolated 1p36 deletions that had been molecularly defined using array-based copy number analysis. Reports of individuals whose 1p36 deletions were not interrogated using such techniques were not included, since traditional cytogenetic techniques may miss copy number changes on other chromosomes, may fail to identify complex rearrangements involving deletion and duplications of various regions of 1p36, and rarely provide robust delineation of the boundaries of the deletion.

Individuals with complex rearrangements of 1p36 that included both deleted and duplicated regions, individuals with 1p36 deletions associated with unbalanced translocations, and individuals with deleterious deletions or duplications on other chromosomes were also excluded from this study.

### Defining Cardiac Critical Regions

Individual cardiac critical regions on chromosome 1p36 were defined based on the smallest, non-overlapping deletion present within a single individual with a cardiovascular malformation and/or cardiomyopathy. In cases where both a minimal and a maximal deleted region were defined, the breakpoints of the maximal deleted region were used to define the critical region. This approach minimizes the risk that a cardiac-related gene located between the minimal and maximal deleted region will be erroneously excluded from the critical region.

### Identifying Cardiac-related Genes within Critical Regions

Data regarding each gene located completely or partially within a cardiac critical region were downloaded into a searchable spreadsheet using GeneDistiller2 (http://www.genedistiller.org/). This publically-available online program allows the user to access information on genes within a defined interval from a variety of online sources. Information reviewed to identify cardiac-related candidate genes included: Online Mendelian Inheritance in Man (OMIM; http://www.omim.org/) reports, interaction data from the Search Tool for the Retrieval of Interacting Genes/Proteins (STRING; http://string-db.org/) and the Unified Human Interactome (UniHI; http://www.unihi.org/), and phenotype data from the Human Phenotype Ontology website (http://www.human-phenotype-ontology.org/), the Mouse Genome Database (MGD; http://www.informatics.jax.org/phenotypes.shtml) and the Gene Ontology Database (http://www.geneontology.org/). This information was augmented with manually curated data from recently published manuscripts cited in PubMed (http://www.ncbi.nlm.nih.gov/pubmed).

## Results

**Figure 1:**
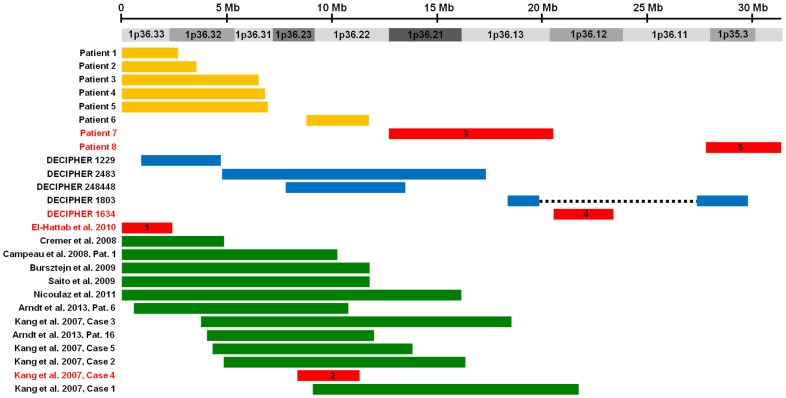
Delineation of five critical regions for cardiovascular malformations on 1p36. Solid bars represent isolated 1p36 deletions identified in individuals with cardiovascular malformations. These deletions are grouped based on the source from which data was obtained: 1) patients referred to the Medical Genetics Laboratories at Baylor College of Medicine for copy number analysis and 2) patients recruited into a research study on 1p36 deletions at Baylor College of Medicine [orange], 3) the DECIPHER database [blue], and 4) a review of the literature [green]. Deletions that define non-overlapping critical region for cardiovascular malformation are shown in red and are numbered sequentially from the telomere.

**Figure 2:**
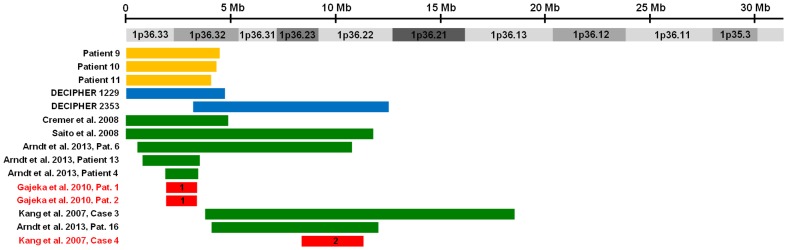
Delineation of two critical regions for cardiomyopathy on 1p36. Solid bars represent isolated 1p36 deletions identified in individuals with cardiomyopathy. These deletions are grouped based on the source from which data was obtained: 1) patients referred to the Medical Genetics Laboratories at Baylor College of Medicine for copy number analysis and 2) patients recruited into a research study on 1p36 deletions at Baylor College of Medicine [orange], 3) the DECIPHER database [blue], and 4) a review of the literature [green]. Deletions that define non-overlapping critical region for cardiomyopathy are shown in red and are numbered sequentially from the telomere.

To map and identify dosage-sensitive genes and genomic regions that may contribute to the development of cardiovascular malformations and/or cardiomyopathy, we identified individuals with these phenotypes who had isolated 1p36 deletions defined by array-based copy number detection techniques as described in the Materials and Methods. The location and extent of the 1p36 deletions identified in each individual are provided in Tables 1–6 along with a description of the individual’s cardiovascular malformations (Tables 1–3) or cardiomyopathy (Tables 4–6). These deletions are also represented graphically in Figures 1 and 2.

**Table 1:** Summary of individuals with isolated 1p36 deletions and cardiovascular malformations referred to the Medical Genetics Laboratory (MGL) at Baylor College of Medicine.

Identifier	Start-Stop(hg19)	Size(Mb)	Heredity	Cardiovascular Malformations	Other	CriticalRegions
Patient 1	1–2694017	2.7	*De novo*	Hypoplastic right heart		1
Patient 2	1–3581432	3.6	*De novo*	Bicommissural aortic valve, mild aorticdilatation	Developmental delay, mild unilateralconductive hearing loss, concern for seizures	1
Patient 3	1–6551698	6.6	*De novo*	Moderate secundum atrial septal defect,dilation of main pulmonary artery	Developmental delay, dysmorphic features,conductive hearing loss	1
Patient 4	1–6804034	6.8	*De novo*	Moderate patent ductus arteriosus, multiplesmall muscular ventricular septal defects,small secundum atrial septal defect, heavilytrabeculated left ventricle with normalfunction		1
Patient 5	1–6921434	6.9	Unknown	Patent ductus arteriosus, multiple ventricularseptal defects, secundum atrial septaldefect, aberrant left subclavian artery	Developmental delay, cognitive impairment,dysmorphic features, intractable seizures,cortical blindness, contractures involvingsmall joints of hand	1
Patient 6	8803013–11739523	2.9	*De novo*	Ventricular septal defect	Microcephaly	2
Patient 7	12726755–20540759	7.8	Unknown	Tetralogy of Fallot,bicommissural aortic valve	Developmental delay, cognitive impairment,seizures, kyphosis of spine	3*
Patient 8	27803719–31404471	3.6	*De novo*	Coarctation of the aorta	Moderate developmental delay, cognitiveimpairment, failure to thrive, gastrointestinalproblems	5*

= Deletion defines a cardiac critical region.

**Table 2:** Summary of individuals with isolated 1p36 deletions and cardiovascular malformations identified from the DECIPHER database.

DECIPHERIdentifier	Start-Stop(hg19)	Size(Mb)	Heredity	CardiovascularMalformations (Co-existing Cardiomyopathy)	Other	CriticalRegions
1229	928301–4708254	3.8	*De novo*	Ventricular septal defect(Cardiomyopathy)	Intellectual disability, epileptic spasms,delayed cranial suture closure, stenosisof external auditory canal, dysmorphic features	1
2483	4795388–17364849	12.6	Not maternal,fatherunavailable	Secundum atrialseptal defect	Intellectual disability, delayed speech andlanguage development, feeding difficultiesin infancy, microcephaly, submucous cleft hard palate,prenatal short stature, scoliosis, dysmorphic features	2, 3
248448	7812397–13488491	5.7	*De novo*	Mild pulmonary valvestenosis	Intellectual disability, feeding difficulties ininfancy, recurrent infections, sensorineuralhearing impairment, proportionate shortstature, cryptorchidism, short phalanges,broad thumbs, dysmorphic features	2, 3
1803 Distal	18382579–19879460	1.5	Unknown	Atrial septal defect	Intellectual disability, feeding difficultiesin infancy, high palate, dysmorphicfeatures	3
1803 Proximal	27358936–29807278	2.4	*De novo*	Atrial septal defect	See above	5
1634	20555776–23438888	2.9	*De novo*	Patent ductus arteriosus,coarctation of the aorta,small ventricular septaldefect	Intellectual disability, behavioralproblems with aggression and mood swings,dysmorphic features, non-cleft velopharyngealdysfunction, hypospadias,bifid thumb, stiffnessand progressive joint contractures withfixed kyphosis, fusion of 1^st^ and 2^nd^cervical vertebrae	4*

= Deletion defines a cardiac critical region.

**Table 3:** Summary of individuals with isolated 1p36 deletions and cardiovascular malformations identified from the literature.

Reference and Patient Identifier	Start-Stop (hg19)	Size (Mb)	Heredity	Cardiovascular Malformations(Co-existing Cardiomyopathy)	Other	CriticalRegions
El-Hattab et al. 2010 [11]	1–2418935	2.4	*De novo*	Ventricular septal defect, atrial septaldefect, PDA, right-sided aortic arch	Developmental delay, renal malpositionand malrotation, omphalocele, cloacalexstrophy, imperforate anus, multiple sacralsegmentation defects, genital anomalies,diastasis of symphysis pubis,limb deformities	1*
Cremer et al.2008 [15]	1–4888723	4.9	*De novo*	Ventricular septal defect (LVNC)	Cleft palate, unilateral choanal stenosis,hypothyroidism	1
Campeau et al. 2008, Patient 1 [16]	1–10247416	10.2	*De novo*	Asymmetric ventricles, muscularventricular septal defect, tortuousaortic arch, PDA	Hypotonia, single febrile seizure, bilateralcolpocephaly, moderate to severenon-obstructive hydrocephalus,sensorineural hearing loss, shortfemurs, unilateral club foot, submucouscleft palate, velopharyngealincompetence, dysmorphic features	1,2
Bursztejn et al. 2009 [17]	1–11809959	11.8	*De novo*	Atrial septal defect, ventricularseptal defect	Infantile spasms, partial seizures,agenesis of the corpus callosum,ventricular dilation,dysmorphic features	1,2
Saito et al. 2008 [18]	1–11809959	11.8	*De novo*	Enlargement of right atrium,narrowing of right ventricle,ventricular septal defects, PDA,Ebstein anomaly (LVNC)	Bilateral perisylvian polymicrogyria,periventricular nodular heterotopia,seizures, hypotonia, feeding difficulties,dysmorphic features	1,2
Nicoulaz et al. 2011 [6]	1–16177338	15.6	*De novo*	Tetralogy of Fallot	Joint contractures, ventriculomegaly,marked pachygyria, absent septumpellucidum, thinned corpus callosum,dysmorphic features	1,2,3
Arndt et al. 2013, Patient 6 [9]	564205–10821909	10.3	*De novo*	Atrial septal defect (LVNC)	Developmental delay, microcephaly,hypotonia, deep set eyes	1,2
Kang et al. 2007, Case 3 [5]	3768946–18563553	14.8	Unknown	Cleft mitral valve, redundanttricuspid valve leaflets,ventricular septal defect, small atrial septal defect,mild pulmonary valvestenosis, PDA	Hypotonia, bilateral nasolacrimal ductobstruction, gastroesophageal reflux,severe biventricular hypertrophy,moderate sensorineural hearing loss,bilateral cleft lip and palate,posteriorly rotated ears,digital contractures	2,3
Arndt et al. 2013, Patient 16 [9]	4089259–12054030	8.0	Unknown	Ventricular septal defect(Cardiomyopathy)	Cognitive impairment,microcephaly, ptosis	2
Kang et al. 2007, Case 5 [5]	4317448–13867316	9.5	Unknown	Partial anomalous pulmonaryvenous return with the leftpulmonary veins draininginto the innominate vein	Developmental delay, seizures,failure to thrive, hemivertebra, Wolff-Parkinson-White syndrome, ataxia,unsteady gait,appendicular hypertonia	2,3
Kang et al. 2007, Case 2 [5]	4843323–16397974	11.6	Unknown	Two small right coronary arteryfistulae terminating in theleft atrium andright ventricle	Developmental delay, seizures,peripheral hypertonia, gastroesophagealreflux, dysmorphic features	2,3
Kang et al. 2007, Case 4 [5]	8395179–11362893	3.0	Unknown	Perimembranous ventricular septaldefect, small septum secundumatrial septal defect	Developmental delay,failure to thrive, truncalhypotonia, dysmorphicfeatures	2*
Kang et al. 2007, Case 1 [5]	9124551–21782714	12.7	Unknown	High and mid muscularventricular septal defect,bicuspid aortic valve,patent foramen ovale, PDA	Developmental delay, seizures,dysmorphic features	2, 3,4

= Deletion defines a cardiac critical region, LVNC = left ventricular noncompaction, PDA = patent ductus arteriosus.

**Table 4:** Summary of individuals with isolated 1p36 deletions and cardiomyopathy referred to the Medical Genetics Laboratory (MGL) or recruited into a 1p36 research study at Baylor College of Medicine.

Identifier	Start-Stop (hg19)	Size (Mb)	Heredity	Cardiomyopathy	Other	Critical Regions
Patient 9	1–4470448	4.5	Unknown	Dilated cardiomyopathy		1
Patient 10	1–4330413	4.3	*De novo*	Dilated cardiomyopathy	Developmental delay, infantile spasms, hypotonia	1
Patient 11	1–4078518	4.1	*De novo*	Bilateral dilatedcardiomyopathy	Seizures, cleft lip, intellectual disability, sensorineural hearing loss	1

**Table 5:** Summary of individuals with isolated 1p36 deletions and cardiomyopathy identified from the DECIPHER database.

DECIPHER Identifier	Start-Stop (hg19)	Size (Mb)	Heredity	Cardiomyopathy (Co-existing Cardiovascular Malformations)	Other	CriticalRegions
1229	1–4708254	4.7	*De novo*	Cardiomyopathy (Ventricular septal defect)	Intellectual disability, development delay, infantile spasms, narrow/atretic auditory canal, dysmorphic features	1
2353	3224674–12540397	9.3	Unknown	Dilated cardiomyopathy	Intellectual disability, development delay, seizures, myopia, sensorineural hearing loss, dysmorphic features	1,2

**Table 6:** Summary of individuals with isolated 1p36 deletions and cardiomyopathy identified from the literature.

Reference and Patient Identifier	Start-Stop (hg19)	Size (Mb)	Heredity	Cardiomyopathy (Co-ExistingCardiovascular Malformations)	Other	CriticalRegions
Cremer et al. 2008 [15]	1–4888723	4.9	*De novo*	Left ventricular noncompaction(Ventricular septal defect)	Large anterior fontanel, cleft palate,right sided choanal stenosis,hypothyroidism	1
Saito et al. 2008 [18]	1–11809959	11.8	*De novo*	Left ventricular noncompaction(Enlargement of right atrium,narrowing of right ventricle,ventricular septal defects,patent ductus arteriosus,Ebstein anomaly)	Bilateral perisylvian polymicrogyria,periventricular nodular heterotopia,seizures, hypotonia, feeding difficulties,dysmorphic features	1,2
Arndt et al. 2013, Patient 6 [9]	564205–10821909	10.3	*De novo*	Left ventricular noncompaction(Atrial septal defect)	Developmental delay,microcephaly, hypotonia, deepset eyes	1
Arndt et al. 2013, Patient 13 [9]	810485–3548853	2.7	*De novo*	Cardiomyopathy	Developmental delay	1
Arndt et al. 2013, Patient 4 [9]	1891455–3465029	1.6	*De novo*	Left ventricular noncompaction	Developmental delay	1
Gajeka et al. 2010, Patient 1 [19]	1916639–3429762	1.5	*De novo*	Left ventricular noncompaction	Developmental delay, moderatesensorineural hearing loss, mildconductive hearing loss, dysmorphicfeatures, hypoglycemia	1*
Gajeka et al. 2010, Patient 2 [19]	1916639–3429762	1.5	*De novo*	Left ventricular noncompaction	Developmental delay, bilateralsensorineural hearing loss,dysmorphic features	1*
Kang et al. 2007, Case 3 [5]	3768946–18563553	14.8	Unknown	Severe biventricular hypertrophy(Cleft mitral valve, redundanttricuspid valve leaflets,ventricular septal defect,small atrial septal defect,mild pulmonary valvestenosis, patent ductus arteriosus)	Hypotonia, bilateral nasolacrimalduct obstruction, gastroesophagealreflux, moderate sensorineuralhearing loss, bilateral cleft lipand palate, posteriorly rotated ears,digital contractures	2
Arndt et al. 2013, Patient 16 [9]	4089259–12054030	8.0	Unknown	Cardiomyopathy (Ventricularseptal defect)	Cognitive impairment,microcephaly, ptosis	2
Kang et al. 2007, Case 4 [5]	8395179–11362893	3.0	Unknown	Dilated cardiomyopathy (Perimembranousventricular septal defect,small septum secundum atrial septal defect)	Developmental delay, failureto thrive, truncal hypotonia,dysmorphic features	2*

= Deletion defines a cardiac critical region.

Using this data, we defined five non-overlapping critical regions for cardiovascular malformations (Table 7, Figure 1). For clarity, each critical region has been numbered sequentially starting with the most distal region on chromosome 1p36. The first critical region is defined by a *de novo* ∼2.4 Mb terminal deletion reported by El-Hattab and colleagues in a patient with a ventricular septal defect, an atrial septal defect, patent ductus arteriosus and a right-sided aortic arch [11].

**Table 7:** Cardiac-related genes within cardiovascular malformation critical regions.

Cardiovascular Malformation Region 1: Chr1∶1–2418935 (2.4 Mb), 111 genes					
Gene	Start (hg19)	Stop (hg19)	Related Cardiovascular Phenotypes		References
*DVL1*	1270658	1284492	No cardiovascular phenotypes have been documented in *Dvl1*-null mice.However, an extra copy of *Dvl1* was able to rescue the lethal conotruncalheart defects seen in *Dvl3*-null mice suggesting that *Dvl1*has redundant functions in cardiac development.		[20], [21]
*SKI*	2160134	2241652	Mutations in *SKI* with putative dominant-negative potential havebeen shown to cause Shprintzen-Goldberg syndrome whose featuresinclude mitral valve prolapse, aortic root dilatation, vascular tortuosityand aortic aneurysms. Knockdown of the 2 paralogs of mammalian*SKI* in zebrafish (*skia* and *skib*) results in severe cardiac anomaliescharacterized by partial to complete failure in cardiac looping andmalformations of the outflow tract.		[22]
**Cardiovascular Malformation Region 2: Chr1∶8395179–11362893 (3.0 Mb), 55 genes**					
*RERE*	8412464	8877699	*Rere*-null mouse embryos die of cardiac failure aroundE9.5 with unlooped hearts. RERE-deficient mouse embryoshave aortic arch anomalies, double outlet right ventricle,transposition of the great arteries, andperimembranous ventricular septal defects.	[23], [24]	
**Cardiovascular Malformation Region 3: Chr1∶12726755–20540759 (7.8 Mb), 175 genes**					
*PDPN*	13910252	13944452	*Pdpn*-null mouse embryos have hypoplastic and perforatedcompact and septal myocardium, hypoplasticatrioventricular cushions resulting in atrioventricularvalve abnormalities, and coronary arteryabnormalities, hypoplasia of the sinoatrial node, andthin, perforated cardinal and pulmonary vein walls.	[25]–[27]	
*SPEN*	16174359	16266950	*Spen*-null mouse embryos die *in utero* and havedefects of the cardiac septum and muscles.	[28]	
*CLCNKA*	16348486	16360545	A loss-of-function variant in the human *CLCNKA*gene is a risk factor for heart failure in Caucasians.	[29]	
**Cardiovascular Malformation Region 4: Chr1∶20555776–23438888 (2.9 Mb), 50 genes**					
*ECE1*	21543740	21672034	A loss-of-function mutation in *ECE1* was identified in anindividual with patent ductus arteriosus, a small subaorticventricular septal defect, and a small atrial-septal defect,Hirschsprung disease, and autonomic dysfunction. *Ece1*-nullmice have interrupted aortic arch, absent right subclavianartery, poorly developed endocardial cushions, doubleoutlet right ventricle, truncus arteriosus, double aortic arch,overriding aorta and ventricular septal defects.	[10], [30], [31]	
*HSPG2*	22148737	22263750	HSPG2-deficient mouse embryos have hyperplasticconotruncal endocardial cushions, transposition of the greatarteries, and malformations of the semilunar valves. However,recessive mutations in *HSPG2* have been shown to causeSchwartz-Jampel syndrome, type 1 and dyssegmental dysplasia,Silverman-Handmaker type, neither of which are commonlyassociated with cardiac defects.	[32]–[34]	
*LUZP1*	23410516	23495351	*Luzp1*-null mice have double outlet right ventricle,transposition of the great arteries, and ventricularseptal defects.	[35]	
**Cardiovascular Malformation Region 5: Chr1∶27803719–31404471 (3.6 Mb), 55 genes**					
*WASF2*	27730734	27816678	*Wasf2*-null mouse embryos die before E11.5 withabnormalities in vasculogenesis. These embryos also havesmall dorsal aortas and anterior cardinal veins at the 22somite stage, and display incomplete cardiac looping andsmall hearts at E10.5.	[36]	

The second critical region is defined by an ∼3.0 Mb interstitial deletion identified in Case 4 reported by Kang and colleagues in an individual with a perimembranous ventricular septal defect and a small septum secundum atrial septal defect [5]. In using Kang et al. Case 4 to define the second critical region, we note that the ∼2.9 Mb interstitial deletion in Patient 6, who had a ventricular septal defect, begins slightly centromeric to the deletion in Kang et al. Case 4 and, therefore, does not involve the *SCL45A1* gene. However, the deletion in Patient 6 extends farther into the proximal region of 1p36 than the deletion in Kang et al. Case 4 and includes *UBE2V2P3*, *PTCHD2*, *FBXO2*, *FBXO44*, *FBXO6* and *MAD2L2*.

The third critical region is defined by an ∼7.8 Mb interstitial deletion in Patient 7 who was referred to the Medical Genetics Laboratories at Baylor College of Medicine for copy number analysis with an indication for testing that included tetralogy of Fallot and a bicommissural aortic valve. The fourth critical region is defined by DECIPHER patient #1634 who had a patent ductus arteriosus, coarctation of the aorta and a small ventricular septal defect caused by a *de novo* ∼2.9 Mb interstitial deletion. The fifth critical region is defined by a *de novo* ∼3.6 Mb interstitial deletion in Patient 8 who was referred to the Medical Genetics Laboratories at Baylor College of Medicine for copy number analysis with an indication for testing that included coarctation of the aorta.

Using this same dataset, we defined two critical regions for cardiomyopathy (Table 8, Figure 2). The first critical region contains the *PRDM16* gene and is defined by an ∼1.5 Mb interstitial deletion in two siblings with left ventricular noncompaction described by Gajeka and colleagues. The second critical region is defined by an ∼3.0 Mb interstitial deletion in an individual reported by Kang and colleagues (Case 4) who had dilated cardiomyopathy which coexisted with a perimembranous ventricular septal defect and a small septum secundum atrial septal defect [5]. Kang and colleagues indicated that both cardiac malformations spontaneously closed but the dilated cardiomyopathy remained and was still being treated with medications when the child was 5 years old. While this history suggests that he had a primary cardiomyopathy, we cannot rule out the possibility that his cardiovascular malformations contributed to his dilated cardiomyopathy. However, further evidence of the role of this region in the development of primary cardiomyopathy comes from Patient 16 described by Arndt and colleagues who had an overlapping ∼8 Mb deletion associated with early-onset cardiomyopathy with transient heart failure whose only structural heart defect was a clinically insignificant ventricular septal defect [9].

**Table 8:** Cardiac-related genes within cardiomyopathy critical regions.

Cardiomyopathy Region 1: Chr1∶1916639–3429762 (1.5 Mb), 28 genes					
Gene	Start (hg19)	Stop (hg19)	Related Cardiovascular Phenotypes		References
*PRKCZ*	1981909	2116834	PRKCZ regulates the phosphorylation and de-phosphorylation ofcardiac sarcomeric proteins including cardiac troponin T. Mutationsin the *TNNT2* gene, which encodes cardiac troponinT, have been found in individuals with leftventricular noncompaction, and dilated, restrictive,and hypertrophic cardiomyopathy.		[37]–[42]
*SKI*	2160134	2241652	In zebrafish, co-injection of subthreshold doses of*skia* and *prdm16* morpholinos reduced cardiac outputsuggesting that these genes act synergistically toaffect cardiac contractility.		[22]
*PRDM16*	2985742	3355185	PRDM16-deficient mice have ventricular hypoplasiaand abnormal ventricular morphology with a cleftbetween ventricles. Morpholino knockdown of*prdm16* in zebrafish causes contractile dysfunction, partialuncoupling of cardiomyocytes and impairedcardiomyocyte proliferative capacity. Deleteriousmutations in *PRDM16* were identified in individualswith left ventricular noncompaction and novel,putatively deleterious sequence variants wereidentified in individuals with dilated cardiomyopathy.		[43], [44]
**Cardiomyopathy Region 2: Chr1∶8395179–11362893 (3.0 Mb), 55 genes**					
*RERE*	8412464	8877699	In the absence of cardiovascular malformations, asubset of RERE-deficient mice spontaneouslydevelops cardiac fibrosis and cardiomegaly.	[24]	
*UBE4B*	10093041	10241297	*Ube4b*-null mice have underdeveloped and compactmyocardial layers, defective assembly of myosin incardiac muscle cells, reduced cardiac trabeculationand cardiac restricted apoptosis that affects mostcardiomyocytes at E13.5.	[45]	
*MASP2*	11086580	11107296	*MASP2* genotypes that generate low MASP2 levelsare associated with a high risk of cardiomyopathy in thesetting of *Trypanosoma cruzi* infection (Chagas disease).However, in a model of transient myocardialischemia/reperfusion injury, *Masp2*-null mice hadsignificantly smaller infarct volumes than their wild-type littermates.	[46], [47]	

Each of the cardiac-related critical regions we identified contains one or more positional candidate genes whose haploinsufficiency may contribute to the development of cardiovascular phenotypes. Genes which may contribute to the development of cardiovascular malformations associated with 1p36 deletions include *DVL1*, *SKI*, *RERE*, *PDPN*, *SPEN*, *CLCNKA*, *ECE1*, *HSPG2*, *LUZP1*, and *WASPF2*. Genes which may contribute to the development of cardiomyopathy associated with 1p36 deletions include *SKI*, *PRKCZ*, *PRDM16, RERE*, *UBE4B* and *MASP2*. The cardiac-related phenotypes associated with each of these genes in humans, mice and zebrafish are summarized in Tables 7 and 8.

## Discussion

Terminal deletions of chromosome 1p36 are the most common telomeric deletions in humans and carry a high risk of cardiac-related medical problems. In one large cohort, 71% of individuals with terminal 1p36 deletions had cardiovascular malformations and 27% had cardiomyopathy [2]. The most common cardiovascular malformations seen were atrial and ventricular septal defects, patent ductus arteriosus, valvular anomalies, tetralogy of Fallot and coarctation of the aorta [2]. Among individuals with cardiomyopathy, 85% had noncompaction defects and the remaining 15% had dilated cardiomyopathy. Similar patterns of cardiac anomalies were seen in the patients with terminal 1p36 deletion described in this report. Individuals with interstitial deletions of 1p36 also have high rates of cardiac abnormalities, although their exact incidences likely vary from one region to another and are more difficult to estimate based on the relatively low number of patients that have been identified [5].

Due to the high risk of cardiac-related problems, most children with 1p36 deletions are screened for cardiovascular anomalies at baseline and followed over time for the development of cardiomyopathy–often having several echocardiograms even if they are asymptomatic. Although this type of careful monitoring can help identify potentially treatable problems, it also places a burden on families and the health care system which could be avoided or reduced if an individual’s risk of having these problems could be estimated based on the location and extent of their individual 1p36 deletion. This type of risk stratification will require a better understanding of the 1p36 genes and genomic regions associated with the development of cardiovascular anomalies and cardiomyopathy.

In this report, we take the first step towards addressing this issue by defining five non-overlapping critical regions for cardiovascular malformations and two non-overlapping critical regions for cardiomyopathy. Each of these cardiac critical regions is defined by an isolated 1p36 deletion identified in a single individual. Hence, they represent genomic regions whose deletions are sufficient to cause cardiovascular malformation or cardiomyopathy. The size of these regions vary with the smallest being 1.5 Mb (28 genes) and the largest being 7.8 Mb (175 genes). As more 1p36 deletions are described, it is likely that these regions will be refined and some may be subdivided into more than one region. It is also possible that data from additional deletions will reveal novel critical regions on 1p36 that are associated with cardiac problems.

It is reasonable to assume that each of the cardiac critical regions we have delineated on chromosome 1p36 contains at least one dosage-sensitive, cardiac-related gene or regulatory region. Although one or more positional candidate genes from each interval can be identified based on their known function and/or their phenotypes in animal models, haploinsufficiency of only two genes have been shown to cause cardiac phenotypes in humans. Heterozygous loss-of-function mutations in *PRDM16* have been shown to be sufficient to cause left ventricular noncompaction and a loss-of-function mutation in *ECE1* was identified in an individual with patent ductus arteriosus, a small subaortic ventricular septal defect and a small atrial septal defect, Hirschsprung disease, and autonomic dysfunction [9], [10].

Loss-of-function mutations in the remainder of the positional candidate genes identified in 1p36 cardiac critical regions–*DVL1*, *SKI*, *RERE*, *PDPN*, *SPEN*, *CLCNKA*, *HSPG2*, *LUZP1*, *WASPF2*, *PRKCZ*, *UBE4B* and *MASP2*–have yet to be identified in individuals with cardiovascular phenotypes. The identification of *de novo* loss-of-function mutations in these positional candidate genes, or dominantly inherited loss-of-function mutations segregating with a cardiac phenotype in multiple family members, would serve to confirm their pathogenic roles. Experience with other chromosomal regions suggests that these types of mutations are most likely to be identified in genes with particularly high impacts on cardiac development and function like *GATA4* on 8p23.1, *ZFPM2* on 8q23.1, and *TBX1* on 22q11.2 [12]–[14].

*De novo* loss-of-function mutations in cardiac genes whose haploinsufficiency makes a more modest contribution to cardiac risk may be particularly difficult to identify. Loss-of-function mutations in such genes are more likely to be inherited from an asymptomatic parent and may combine with other genetic, environmental or stochastic factors to cause cardiac-related problems in sporadic cases following a multifactorial inheritance pattern. In such cases, animal models may provide the first evidence that a gene within one of the critical regions plays a role in cardiac development and/or function. Not only can animal models be used to effectively identify low penetrance phenotypes caused by haploinsufficiency of a candidate gene, but they can also be used to explore cardiac-related phenotypes–including defects that lead to embryonic lethality–that only become apparent when the expression of an individual gene is reduced by more than 50%.

Deleterious changes in genes that make a modest contribution to cardiac risk may also be identified in asymptomatic individuals from the general population. One source of information on potentially deleterious changes in various populations is the Database of Genomic Variants (http://dgv.tcag.ca/) which catalogues genomic variation among “normal controls” and population-based cohorts. It is interesting to note that exon-containing deletions in each of the positional candidate genes identified in this study have been reported in this database (Table S1). This suggests that the cardiac-related phenotypes caused by haploinsufficiency of each of these genes alone are likely to be incompletely penetrant or may go undetected in some individuals. This is consistent with the incomplete penetrance for cardiovascular malformations and cardiomyopathy seen among patients with overlapping terminal and interstitial deletions of 1p36 [2], [5].

Several of the cardiac critical regions we have identified on chromosome 1p36 contain more than one positional candidate gene. We also note that large terminal and interstitial deletions often overlap more than one cardiac critical region. This suggests that haploinsufficiency of two or more genes may contribute to the cardiac phenotypes associated with many 1p36 deletions. Future studies aimed at understanding how adjacent 1p36 genes work together to impact cardiac development and function may provide information which can be used to design effective therapeutic and/or preventative measures which can minimize the impact of cardiovascular malformations and/or cardiomyopathy in such cases.

## Supporting Information

Table S1 **Exon-containing deletions in 1p36 cardiac-related genes found in various control and population-based cohorts catalogued in the Database of Genomic Variants.**
(DOC)
