# Supplementary material for: Identification of Critical Regions and Candidate Genes for Cardiovascular Malformations and Cardiomyopathy Associated with Deletions of Chromosome 1p36
Source: PLoS One. 2014 Jan 15;9(1):e85600. doi: 10.1371/journal.pone.0085600 (PMC3893250; doi:10.1371/journal.pone.0085600)
Supplement: Table S1 — Exon-containing deletions in 1p36 cardiac-related genes found in various control and population-based cohorts catalogued in the Database of Genomic Variants. (DOC) [file pone.0085600.s001.doc]

**Table S1.** Exon-containing deletions in 1p36 cardiac-related genes found in various control and population-based cohorts catalogued in the Database of Genomic Variants

| **Gene*** | **Reference** | **Exon containing deletions/Sample size (Ratio)** | **Population description** |
| --- | --- | --- | --- |
| *DVL1* | Xu et al. 2011 [1] | 69/6533 (0.0106) | Randomly selected individuals from three population cohorts residing in Singapore: 1917 Chinese, 2399 Malays, 2217 Indians |
| *DVL1* | Jakobsson et al. 2008 [2] | 3/443 (0.0068) | World-wide sample of individuals from the Human Genome Diversity-Centre d'Etude du Polymorphisme Humain (HGDP-CEPH) panel |
| *DVL1* | Iafrate et al. 2004 [3] | 1/39 (0.0256) | Unrelated health control individuals with normal karyotypes |
| *SKI* | Xu et al. 2011 [1] | 57/6533 (0.0087) | Randomly selected individuals from three population cohorts residing in Singapore: 1917 Chinese, 2399 Malays, 2217 Indians |
| *SKI* | Itsara et al. 2009 [4] | 1/1557 (0.0006) | 671 individuals of European descent with no family history of or any first-degree relative with amyotrophic lateral sclerosis, ataxia, autism, brain aneurysm, dystonia, Parkinson disease, or schizophrenia and 886 individuals sampled from 51 different world populations included in the Human Genome Diversity Panel (HGDP) |
| *SKI* | Jakobsson et al. 2008 [2] | 8/443 (0.0181) | World-wide sample of individuals from the Human Genome Diversity-Centre d'Etude du Polymorphisme Humain (HGDP-CEPH) panel |
| *SKI* | McKernan et al. 2009 [5] | 1/1 | Anonymous male from Yoruba in Ibadan, Nigeria who is part of the International HapMap Project |
| *SKI* | Wong et al. 2007 [6] | 59/95 (0.6211) | 14 healthy blood donors, 51 individuals from a British Columbia Cancer Agency screening program, 16 samples from the Human Variation Collection and 14 CEPH pedigree samples |
| *RERE* | Shaikh et al. 2009 [7] | 9/2026 (0.0044) | Healthy subjects routinely seen at primary care and well-child clinic practices within the Children’s Hospital of Philadelphia (CHOP) Health Care Network: 1320 Caucasians, 694 African Americans and 12 Asian Americans |
| *RERE* | Xu et al. 2011 [1] | 3/6533 (0.0005) | Randomly selected individuals from three population cohorts residing in Singapore: 1917 Chinese, 2399 Malays, 2217 Indians |
| *RERE* | Park et al. 2010 [8] | 1/31 (0.0323) | 10 Korean females, 10 HapMap Chinese females, 10 HapMap Japanese females and a reference sample (NA10851) |
| *RERE* | Conrad et al. 2009 [9] | 12/39 (0.3077) | 39 HapMap females: 20 from Utah with northern and western European ancestry and 19 Yoruba females from Ibadan, Nigeria |
| *RERE* | Matsuzaki et al. 2009 [10] | 9/90 (0.1000) | 90 HapMap Yoruba individuals from Ibadan, Nigeria who were members of 30 family trios |
| *PDPN* | Itsara et al. 2009 [4] | 1/1557 (0.0006) | 671 individuals of European descent with no family history of or any first-degree relative with amyotrophic lateral sclerosis, ataxia, autism, brain aneurysm, dystonia, Parkinson disease, or schizophrenia and 886 individuals sampled from 51 different world populations included in the Human Genome Diversity Panel (HGDP) |
| *SPEN* | Xu et al. 2011 [1] | 2/6533 (0.0003) | Randomly selected individuals from three population cohorts residing in Singapore: 1917 Chinese, 2399 Malays, 2217 Indians |
| *SPEN* | Kidd et al. 2008 [11] | 1/1 | One control sample (NA12156) from the Centre d'Etude du Polymorphisme Humain (CEPH) collection |
| *CLCNKA* | Xu et al. 2011 [1] | 13/6533 (0.0020) | Randomly selected individuals from three population cohorts residing in Singapore: 1917 Chinese, 2399 Malays, 2217 Indians |
| *CLCNKA* | Shaikh et al. 2009 [7] | 10/2026 (0.0049) | Healthy subjects routinely seen at primary care and well-child clinic practices within the Children’s Hospital of Philadelphia (CHOP) Health Care Network: 1320 Caucasians, 694 African Americans and 12 Asian Americans |
| *CLCNKA* | Ahn et al. 2009 [12] | 1/1 | Korean male genome donor (Seong-Jin Kim) from the Korean reference genome construction project |
| *ECE1* | Xu et al. 2011 [1] | 2/6533 (0.0003) | Randomly selected individuals from three population cohorts residing in Singapore: 1917 Chinese, 2399 Malays, 2217 Indians |
| *ECE1* | Pang et al. 2010 [13] | 1/1 | Individual male HuRef (J. Craig Venter) |
| *HSPG2* | Gusev et al. 2009 [14] | 1/2906 (0.0003) | Non-selected adults from Kosrae, Micronesia |
| *HSPG2* | Itsara et al. 2009 [4] | 3/1557 (0.0019) | 671 individuals of European descent with no family history of or any first-degree relative with amyotrophic lateral sclerosis, ataxia, autism, brain aneurysm, dystonia, Parkinson disease, or schizophrenia and 886 individuals sampled from 51 different world populations included in the Human Genome Diversity Panel (HGDP) |
| *HSPG2* | Xu et al. 2011 [1] | 13/6533 (0.0020) | Randomly selected individuals from three population cohorts residing in Singapore: 1917 Chinese, 2399 Malays, 2217 Indians |
| *HSPG2* | Locke et al. 2006 [15] | 2/265 (0.0075) | European, Yoruba, Chinese, and Japanese individuals from the International HapMap Consortium |
| *HSPG2* | Wong et al. 2007 [6] | 1/95 (0.0105) | 14 healthy blood donors, 51 individuals from a British Columbia Cancer Agency screening program, 16 samples from the Human Variation Collection and 14 CEPH pedigree samples |
| *HSPG2* | Pang et al. 2010 [13] | 1/1 | Individual male HuRef (J. Craig Venter) |
| *HSPG2* | Jakobsson et al. 2008 [2] | 2/443 (0.0045) | World-wide sample of individuals from the Human Genome Diversity-Centre d'Etude du Polymorphisme Humain (HGDP-CEPH) panel |
| *LUXP1* | Xu et al. 2011 [1] | 1/6533 (0.0002) | Randomly selected individuals from three population cohorts residing in Singapore: 1917 Chinese, 2399 Malays, 2217 Indians |
| *WASF2* | Xu et al. 2011 [1] | 4/6533 (0.0006) | Randomly selected individuals from three population cohorts residing in Singapore: 1917 Chinese, 2399 Malays, 2217 Indians |
| *WASF2* | Itsara et al. 2009 [4] | 2/1557 (0.0013) | 671 individuals of European descent with no family history of or any first-degree relative with amyotrophic lateral sclerosis, ataxia, autism, brain aneurysm, dystonia, Parkinson disease, or schizophrenia and 886 individuals sampled from 51 different world populations included in the Human Genome Diversity Panel (HGDP) |
| *WASF2* | Shaikh et al. 2009 [7] | 2/2026 (0.0010) | Healthy subjects routinely seen at primary care and well-child clinic practices within the Children’s Hospital of Philadelphia (CHOP) Health Care Network: 1320 Caucasians, 694 African Americans, 12 Asian Americans |
| *PRKCZ* | Xu et al. 2011 [1] | 31/6533 (0.0047) | Randomly selected individuals from three population cohorts residing in Singapore: 1917 Chinese, 2399 Malays, 2217 Indians |
| *PRKCZ* | Itsara et al. 2009 [4] | 2/1557 (0.0013) | 671 individuals of European descent with no family history of or any first-degree relative with amyotrophic lateral sclerosis, ataxia, autism, brain aneurysm, dystonia, Parkinson disease, or schizophrenia and 886 individuals sampled from 51 different world populations included in the Human Genome Diversity Panel (HGDP) |
| *PRKCZ* | Jakobsson et al. 2008 [2] | 1/443 (0.0023) | World-wide sample of individuals from the Human Genome Diversity-Centre d'Etude du Polymorphisme Humain (HGDP-CEPH) panel |
| *PRKCZ* | Wong et al. 2007 [6] | 11/95 (0.1158) | 14 healthy blood donors, 51 individuals from a British Columbia Cancer Agency screening program, 16 samples from the Human Variation Collection and 14 CEPH pedigree samples |
| *PRDM16* | Xu et al. 2011 [1] | 49/6533 (0.0075) | Randomly selected individuals from three population cohorts residing in Singapore: 1917 Chinese, 2399 Malays, 2217 Indians |
| *PRDM16* | Itsara et al. 2009 [4] | 2/1557 (0.0013) | 671 individuals of European descent with no family history of or any first-degree relative with amyotrophic lateral sclerosis, ataxia, autism, brain aneurysm, dystonia, Parkinson disease, or schizophrenia and 886 individuals sampled from 51 different world populations included in the Human Genome Diversity Panel (HGDP) |
| *PRDM16* | Jakobsson et al. 2008 [2] | 10/443 (0.0226) | World-wide sample of individuals from the Human Genome Diversity-Centre d'Etude du Polymorphisme Humain (HGDP-CEPH) panel |
| *PRDM16* | Wong et al. 2007 [6] | 20/95 (0.2105) | 14 healthy blood donors, 51 individuals from a British Columbia Cancer Agency screening program, 16 samples from the Human Variation Collection and 14 CEPH pedigree samples |
| *UBE4B* | McKernan et al. 2009 [5] | 1/1 | Anonymous male from Yoruba in Ibadan, Nigeria who is part of the International HapMap Project |
| *MASP2* | Xu et al. 2011 [1] | 2/6533 (0.0003) | Randomly selected individuals from three population cohorts residing in Singapore: 1917 Chinese, 2399 Malays, 2217 Indians |
| *MASP2* | Conrad et al. 2009 [9] | 12/450 (0.0267) | 450 HapMap individuals: 180 individuals from Utah with northern and western European ancestry, 180 Yoruba individuals from Ibadan, Nigeria, 45 Japanese individuals from Tokyo, Japan and 45 Chinese individuals from Beijing, China |

* The Database of Genomic Variants was accessed on 8-6-2013

**References**

1. Xu H, Poh WT, Sim X, Ong RT, Suo C, et al. (2011) SgD-CNV, a database for common and rare copy number variants in three Asian populations. Hum Mutat 32: 1341-1349.

2. Jakobsson M, Scholz SW, Scheet P, Gibbs JR, VanLiere JM, et al. (2008) Genotype, haplotype and copy-number variation in worldwide human populations. Nature 451: 998-1003.

3. Iafrate AJ, Feuk L, Rivera MN, Listewnik ML, Donahoe PK, et al. (2004) Detection of large-scale variation in the human genome. Nat Genet 36: 949-951.

4. Itsara A, Cooper GM, Baker C, Girirajan S, Li J, et al. (2009) Population analysis of large copy number variants and hotspots of human genetic disease. Am J Hum Genet 84: 148-161.

5. McKernan KJ, Peckham HE, Costa GL, McLaughlin SF, Fu Y, et al. (2009) Sequence and structural variation in a human genome uncovered by short-read, massively parallel ligation sequencing using two-base encoding. Genome Res 19: 1527-1541.

6. Wong KK, deLeeuw RJ, Dosanjh NS, Kimm LR, Cheng Z, et al. (2007) A comprehensive analysis of common copy-number variations in the human genome. Am J Hum Genet 80: 91-104.

7. Shaikh TH, Gai X, Perin JC, Glessner JT, Xie H, et al. (2009) High-resolution mapping and analysis of copy number variations in the human genome: a data resource for clinical and research applications. Genome Res 19: 1682-1690.

8. Park H, Kim JI, Ju YS, Gokcumen O, Mills RE, et al. (2010) Discovery of common Asian copy number variants using integrated high-resolution array CGH and massively parallel DNA sequencing. Nat Genet 42: 400-405.

9. Conrad DF, Pinto D, Redon R, Feuk L, Gokcumen O, et al. (2010) Origins and functional impact of copy number variation in the human genome. Nature 464: 704-712.

10. Matsuzaki H, Wang PH, Hu J, Rava R, Fu GK (2009) High resolution discovery and confirmation of copy number variants in 90 Yoruba Nigerians. Genome Biol 10: R125.

11. Kidd JM, Cooper GM, Donahue WF, Hayden HS, Sampas N, et al. (2008) Mapping and sequencing of structural variation from eight human genomes. Nature 453: 56-64.

12. Ahn SM, Kim TH, Lee S, Kim D, Ghang H, et al. (2009) The first Korean genome sequence and analysis: full genome sequencing for a socio-ethnic group. Genome Res 19: 1622-1629.

13. Pang AW, MacDonald JR, Pinto D, Wei J, Rafiq MA, et al. (2010) Towards a comprehensive structural variation map of an individual human genome. Genome Biol 11: R52.

14. Gusev A, Lowe JK, Stoffel M, Daly MJ, Altshuler D, et al. (2009) Whole population, genome-wide mapping of hidden relatedness. Genome Res 19: 318-326.

15. Locke DP, Sharp AJ, McCarroll SA, McGrath SD, Newman TL, et al. (2006) Linkage disequilibrium and heritability of copy-number polymorphisms within duplicated regions of the human genome. Am J Hum Genet 79: 275-290.
